# Phytotoxic Activities of Mediterranean Essential Oils

**DOI:** 10.3390/molecules15064309

**Published:** 2010-06-14

**Authors:** Luiz Fernando Rolim de Almeida, Fernando Frei, Emilia Mancini, Laura De Martino, Vincenzo De Feo

**Affiliations:** 1 Departamento de Botânica, Instituto de Biociências de Botucatu, UNESP - Campu de Botucatu Distrito de Rubião Júnior, S/N, 18.618-000, Botucatu-SP, Brazil; E-Mail: rolimdealmeidalf@yahoo.com.br (L.F.R.A.); 2 Departamento de Ciências Biológicas, Faculdade de Ciências e Letras, UNESP – Universidade Estadual Paulista, Avenida Dom Antonio, 19806-900, Assis-SP, Brazil; E-Mail: botanica@ibb.unep.br (F.F.); 3 Dipartimento di Scienze Farmaceutiche, Università degli Studi di Salerno, via Ponte Don Melillo, 84084 Fisciano (Salerno), Italy; E-Mails: emancini@unisa.it (E.M); ldemartino@unisa.it (L.D.M.)

**Keywords:** essential oils, phytotoxicity, germination, seedling growth, monoterpenes

## Abstract

Twelve essential oils from Mediterranean aromatic plants were tested for their phytotoxic activity, at different doses, against the germination and the initial radicle growth of seeds of *Raphanus sativus*, *Lactuca sativa* and *Lepidium sativum*. The essential oils were obtained from *Hyssopus officinalis*, *Lavandula angustifolia*, *Majorana hortensis*, *Melissa officinalis*, *Ocimum basilicum*, *Origanum vulgare*, *Salvia officinalis* and *Thymus vulgaris* (Lamiaceae), *Verbena officinalis* (Verbenaceae), *Pimpinella anisum*, *Foeniculum vulgare* and *Carum carvi* (Apiaceae). The germination and radicle growth of tested seeds were affected in different ways by the oils. Thyme, balm, vervain and caraway essential oils were more active against both germination and radicle elongation.

## 1. Introduction

Potential damage to human health and to the environment provoked by synthetic herbicides is regarded today as a real problem. It has resulted in an increased interest in alternative strategies leading to the development of biodegradable and non-toxic compounds [1]. In fact, the continued use of synthetic herbicides may threaten sustainable agricultural production and result in serious ecological and environmental problems, such as the increased resistance of weeds and environmental pollution and health hazards [2]. Volatile oils and their constituents are being explored for weed and pest management and are viewed as an important source of lead molecules in agriculture [3]. It is thus pertinent to explore and characterize the phytotoxic properties of aromatic plants and their volatile oils. Bioactive terpenoids constitute an important part of the defensive mechanisms of a large number of organisms and represent a fairly untapped source of active compounds of potential use both in the agricultural and pharmaceutical fields [4]. In fact, a large number of highly phytotoxic allelochemicals are derived from the terpenoid pathway [5] and the phytotoxicity of essential oils has been investigated in various plant tissues which contains or produce these compounds [2,6,7,8]. The Mediterranean flora is characterized by the abundance of aromatic plants among its components. The feature differentiating these plants from all others, in spite of the fact that they belong to many different families, is the production of chemically related secondary compounds, the low molecular weight and volatile isoprenoids. This remarkable presence of aromatic species is important in determining the allelopathic potential within this ecosystem [9]. Thus, the objective of this study was to evaluate the *in vitro* possible phytotoxicity of the essential oils from 12 Mediterranean plants, belonging to three different families, *Hyssopus officinalis* L. (hyssop), *Lavandula angustifolia* Mill. (lavender), *Majorana hortensis* L. (marjoram), *Melissa officinalis* L. (lemon balm), *Ocimum basilicum* L. (basil), *Origanum vulgare* L. (oregano), *Salvia officinalis* L. (sage), *Thymus vulgaris* L. (thyme) (Lamiaceae), *Carum carvi* L. (caraway), *Foeniculum vulgare* Mill. (fennel), *Pimpinella anisum* L. (anise) (Apiaceae), *Verbena officinalis* L. (vervain) (Verbenaceae) against the germination and radicle growth of the crop species *Raphanus sativus* L. cv. Saxa (radish), *Lepidium sativum* L. (garden cress) and *Lactuca sativa* L. (lettuce), comparing the effects of the oils in light of their chemical composition.

## 2. Results and Discussion

The yields in essential oil obtained by hydrodistillation of plant species collected at full flowering stage, on a fresh weight basis, were as follows: *H. officinalis* 0.41%, *L. angustifolia* 0.49%, *M. hortensis* 0.26%, *M. officinalis* 0.25%, *O. basilicum* 0.42%, *O. vulgare* 0.21%, *S. officinalis* 0.46%, *T. vulgaris* 0.26%, *V. officinalis* 0.39%, *P. anisum* 1.80%, *F. vulgare* 2.30% and *C. carvi* 2.80%. 

Table 1 shows the composition of the essential oils. The main constituent of *P. anisum* and *F. vulgare* essential oils was *cis*-anethole, which represented 97.1% and 76.3% of the whole oils, respectively. Our data on anise oil composition agree with the available literature. Tabanca and coworkers [10] reported that anise oil was constituted predominantly by anethole (94.2%). *F. vulgare* oil is also reported to contain mainly anethole [11]. The dominant components in *C. carvi* oil were estragole (65.0%), limonene (14.3%), β-pinene (7.4%) and *trans*-pinocamphone (4.3%). Limonene and carvone were reported as the main components [12] of caraway oil and also our study confirmed limonene as one of the most abundant components of this oil. Vervain essential oil was mainly constituted by citral and isobornyl formate. A previous study reported a different composition for vervain oil: Ardakani and coworkers [13] identified 3-hexen-1-ol, 1-octen-3-ol, linalol, verbenone and geranial as its major components. In general, the composition of Lamiaceae oils agrees with the available literature. β-Pinene (18.2%), *iso-*pinocamphone (29.1%), and *trans*-pinocamphone (11.2%) were the most abundant components of *H. officinalis* essential oil. This composition agrees with the available literature [14]. Linalol (23.1%) and linalyl acetate (44.4%) represented the main components of the oil of *L. angustifolia*; also in this case the composition is similar to data reported in literature [15]. Marjoram essential oil was mainly constituted by 1,8-cineole (33.5%), α-pinene (9.0%) and limonene (6.4%). The main constituents of *M. officinalis* essential oil were (-)-citronellal (39.6%), carvacrol (13.3%) and *iso*-menthone (8.8%); *iso*-pinocamphone (35.1%) and carvone (39.7%) were the predominant components of *O. basilicum* essential oil. The compositions of the latter oils agree with literature data [16,17]. In *O. vulgare* and *T. vulgaris* oils, *o*-cymene and carvacrol were the main constituents, accounting, respectively, for 41.9% and 44.0%, in oregano, and 56.2% and 24.4% in thyme oil. The oregano oil appears to be in part different from others reported in literature: in fact, some papers reported *p*-cymene as the main compound of this oil [18]. Differences were also reported for the composition of thyme oil [2,16]. Sage essential oil was mainly constituted by *trans*-thujone (37.9%), camphor (13.9%) and borneol (7.6%) and this composition agrees with literature reports [19]. 

A comparison among the chemical groups present in essential oils was performed in order to verify the similarity of the oil composition among the different plants (Table 2). The components of the oils were divided into 5 chemical groups: alcohols, aldehydes, alkenes, ketones and phenols. Balm and vervain oils were characterized by a high presence of aldehydes, about 39% and 44% of the total oil, respectively. Lavender was characterized by a strong presence of alcohols (41.1%), while marjoram was characterized by a high presence of alkenes (14.8%). Thyme and oregano oils belong to the same group, as determined by the presence of phenols, that represent about 33.1% and 44.8% of the total oil composition, respectively. Hyssop, sage and basil oils belong to the group characterized by the presence of ketones (about 41%, 76% and 52%, respectively). Finally, caraway was characterized by the presence of estragole, while the other two apiaceous oils (fennel and anise) are characterized by the high presence of anethole (77.1% and 97.1%, respectively). 

Monoterpenes were the most abundant components of all the oils analysed, except for the fennel and anise oils, representing a percentage ranging between 82.3%, in the hyssop oil and 97.4%, in the oil of thyme. Among monoterpenes, oxygenated compounds were in amounts ranging between 47.4% (oregano oil) and 91.2% (vervain). Sesquiterpenes were in lower amounts in all the oils. On the other hand, the oils of anise and fennel were mainly constituted of non terpenes ranging between 97.1%, in the anise oil, and 76.3%, in fennel. 

In general, a high presence of oxygenated monoterpenes is linked to a potent phytotoxic activity [20]. Vokou and coworkers [21] studied the allelopathic activities of 47 monoterpenoids belonging to different chemical groups, estimating their effects on seed germination and subsequent growth of *Lactuca sativa* seedlings and found that the most active compounds against both processes belonged to the groups of ketones and alcohols, followed by the group of aldehydes and phenols. Our data agree with this finding: all oils were active against germination and early radicle growth of *Lepidium sativum, Raphanus sativus* and *Lactuca sativa,* but at different levels of activity (Table 3, Table 4 and Table 5).

**Figure 1:**
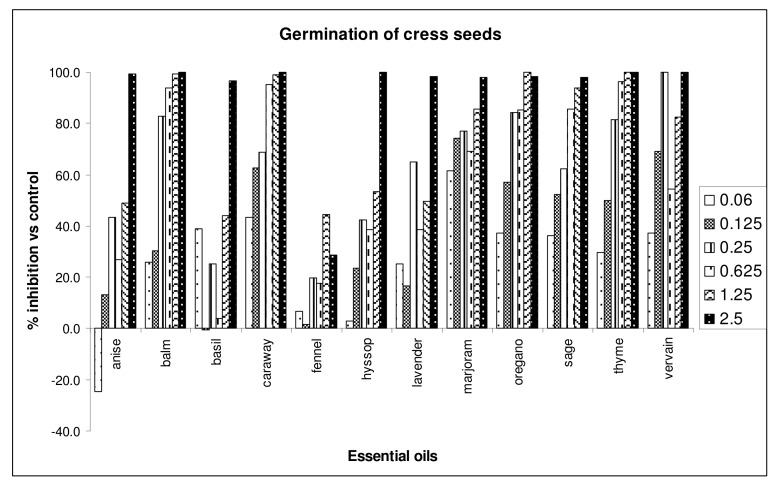
Percent inhibition of germination of *Lepidium sativum* seeds treated with different doses of essential oils.

The germination of *Lepidium sativum* was drastically affected by a 2.5 μg/mL dose of the essential oils of balm, caraway, hyssop, thyme and vervain, with a 100% inhibition (Figure 1). 

**Figure 2:**
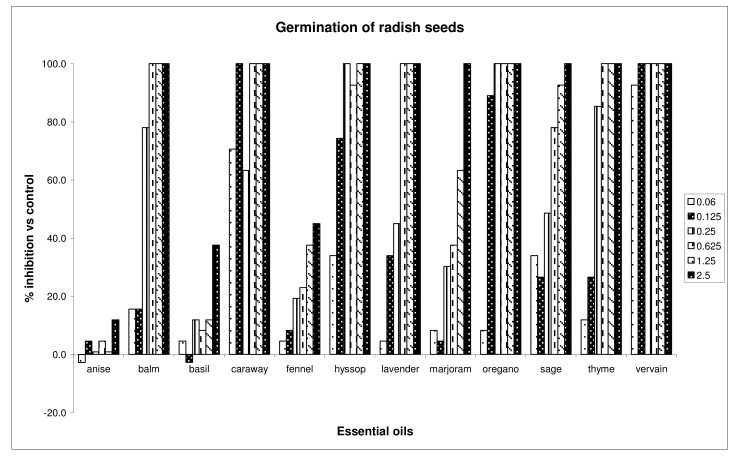
Percent inhibition of germination of *Raphanus sativus* seeds treated with different doses of essential oils.

Thyme and oregano oils inhibited both germination and radicle elongation at a dose of 1.25 μg/mL. Caraway, vervain, sage and marjoram essential oils affected, in a significative way, the radicle elongation of this seed, at all doses. Anise oil was the less active on germination, whereas fennel oil was less active on radicle elongation of garden cress. Moreover, some oils (anise, basil), at the lowest dose, promoted the germination and/or radicle elongation of garden cress. Generally, garden cress is the less sensitive seed. Almost all oils, except anise, basil and fennel, inhibited by 100% the germination of *R. sativus*, at the highest dose tested (Figure 2). 

**Figure 3:**
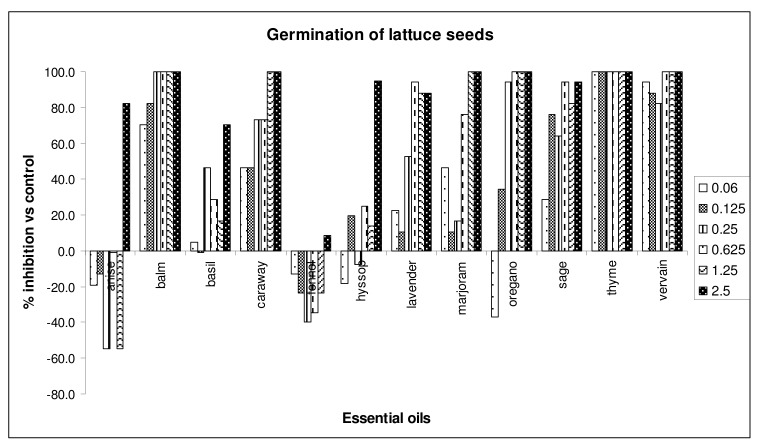
Percent inhibition of germination of *Lactuca sativa* seeds treated with different doses of essential oils.

Vervain oil inhibited by 100% the germination of radish, at almost all doses tested. In addition, caraway, hyssop and sage oils inhibited, in a significative way, the germination of radish, at all doses tested. The radicle growth of the same seeds was affected by 100% by vervain, caraway, oregano, thyme, hyssop and lavender essential oils, at the three highest doses assayed (0.625, 1.25 and 2.5 μg/mL). Moreover, all oils cited above, except lavender, were active towards radicle elongation, at all doses. The germination and radicle elongation of *Lactuca sativa* were affected by vervain, balm, caraway and oregano oils, resulting in a maximum inhibitory activities (100% inhibition), at doses of 1.25 and 2.5 μg/mL (Figure 3). 

Thyme oil inhibited by 100% germination and radicle elongation of lettuce seeds, at all assayed doses. Moreover, vervain, balm and caraway oils inhibited, significantly, germination of lettuce seeds, at all doses. Marjoram and vervain, and caraway again, inhibited, in a significative way, the radicle growth of the seeds. Also in this case, fennel and anise oils were among less active ones, against both germination and radicle elongation.

As reported in this paper, some essential oils possess strong phytotoxic effect; this opens the door to their use as herbicides. Although few studies have addressed this herbicidal activity, the authors in [22] demonstrated that some essential oils, including thyme oil, are highly phytotoxic [23]. Kordali and coworkers [24] reported that the herbicidal effects of oregano oil can be attributed to its major component, carvacrol. Moreover, it has been documented that some essential oils isolated and their phenolic compounds, carvacrol and thymol, possess potent herbicidal effects on weed germination and seedling growth of various plant species [2,25]. 

Some doses of the same oil are inhibitory, other stimulatory. The concept of a generalized “low-dose stimulation - high-dose inhibition” or “hormesis” was gradually supported by field observations [26]. There is evidence that exposure to novel environments or a toxic substance increases the variance of phenotypic traits such as enzyme activity [27], morphological features [28] and growth [29]. However, the reasons for such increases and their adaptive implications remain unclear [30].

Dudai and coworkers [31] reported that monoterpenes act on seeds at very low levels, and that their content in various parts of wheat seeds differs. In particular, among the Lamiaceae family, many species release phytotoxic monoterpenes that hinder the development of herbaceous species among which β-pinene, limonene, *p*-cymene, 1,8-cineole [2]. In previous studies, plants exposed to monoterpene vapour have shown severe internal damage. The absence of a variety of intact organelles and the presence of membrane fragments indicate that structural breakdown and decomposition occur within inhibited roots [32]. Some of the most inhibitory compounds have been repeatedly reported as phytotoxic against a number of target species [33,34], though not always with the same level of activity. Reynolds [34], also working with *L. sativa*, compared a large number of compounds belonging to different chemical groups as to their effect on seed germination and early seedling development. 

Moreover, it is well known that monoterpenes in the essential oils have phytotoxic effects that may cause anatomical and physiological changes in plant seedlings leading to accumulation of lipid globules in the cytoplasm, reduction in some organelles such as mitochondria, possibly due to inhibition of DNA synthesis or disruption of membranes surrounding mitochondria and nuclei [35,36]. 

## 3. Experimental Section

### 3.1. Plant material

Plants of *Hyssopus officinalis*, *Lavandula angustifolia*, *Majorana hortensis*, *Melissa officinalis*, *Ocimum basilicum*, *Origanum vulgare*, *Salvia officinalis* and *Thymus vulgaris*, *Verbena officinalis*, *Pimpinella anisum*, *Foeniculum vulgare* and *Carum carvi* were grown at the Garden of Medicinal Plants in Salerno, State University Campus. Samples from the above plant species were collected at full flowering stage, in July-August 2008. Vouchers specimens of each plant were deposited in the herbarium of the Medical Botany Chair, Faculty of Pharmacy, Salerno University.

### 3.2. Oil isolation

Five-hundred g of freshly picked aerial parts of each lamiaceous species, aerial parts of vervain, and fruits of each apiaceous species, were cut into small pieces and then subjected to hydrodistillation for 3 h, following the procedure described in the European Pharmacopoeia [37]. Extraction procedure was repeated three times, on three samples of the same drug.

### 3.3. GC and GC-MS analyses

Essential oils were analysed by gas chromatography (GC) and gas chromatography-mass spectrometry (GC-MS). GC analyses were performed using a Perkin-Elmer Sigma-115 gas chromatograph with a data handling system and a FID. Analyses were carried out using a DB-1 fused-silica column (30 m × 0.25 mm i.d; 0.25 μm film thickness). The operating conditions were as follows: injector and detector temperatures, 250 and 280 ºC, respectively; oven temperature programme, 5 min isothermal at 40 ºC, then at 2 ºC/min up to 250 ºC and finally held isothermally for 20 min. Aliquots of 1 μL were injected manually at 250 ºC and in the splitless mode. Analysis was also run by using a fused silica HP Innowax polyethylene glycol capillary column (50 m × 0.20 mm i.d.; 0.20 μm film thickness). In both cases, helium was used as the carrier gas (1 mL/min). Diluted samples (1/100 v/v, in *n-*hexane) of 1 μL were injected manually at 250 ºC and in the splitless mode. GC–MS analyses were carried out using a Hewlett-Packard 5890A gas chromatograph connected on line to a HP Mass Selective Detector (MSD 5970 HP), equipped with a HP-1 fused-silica column (25 m × 0.25 mm i.d.; 0.33 μm film thickness); GC and GC-MS analyses: ionization voltage 70; electron multiplier energy 2000 V. Gas chromatographic conditions were as reported above; transfer line was kept at 295 ºC. Most components of the essential oils were identified on the basis of their GC retention indices or of their MS spectra that were compared either with those reported in literature [38,39] either with those stored in NBS and Wiley5 libraries or with those of standard compounds available in our laboratories and purchased from Sigma Aldrich, Co. Milan, Italy. The retention indices were determined in relation to a homologous series of *n*-alkanes (C_8_-C_24_) under the same operating conditions. Relative concentrations of each essential oil component were calculated on the basis of GC peaks without using correction factors.

### 3.4. Biological assay

A bioassay based on germination and subsequent radicle growth was used to study the phytotoxic effects of the essential oils on seeds of *Raphanus sativus*, *Lactuca sativa* and *Lepidium sativum* L. The seeds were surface-sterilized in 95% ethanol for 15 s and sown in Petri dishes (Ø = 90 mm), containing five layers of Whatman filter paper, impregnated with distilled water (7 mL, control) or tested solution of the essential oil (7 mL), at the different assayed doses. The essential oils, in water–acetone mixture (99.5:0.5), were assayed at the follow doses: 2.5, 1.25, 0.625, 0.25, 0.125 and 0.06 μg/mL. Controls performed with water–acetone mixture alone showed no appreciable difference in comparison with controls in water alone. The germination conditions were as follow: for radish and garden cress seeds, 20 ± 1 ºC, and for lettuce seeds, 24 ± 1 ºC, with natural photoperiod. Seed germination process was observed directly in Petri dishes, each 24 hours. A seed was considered germinated when the protrusion of the root became evident [40]. After 96 h (on the fourth day), the effects on radicle elongation were measured (the lengths were measured in centimeters). Each determination was repeated three times, using Petri dishes containing 10 seeds each. Data are expressed as the mean ± SE of both germination and radicle elongation.

### 3.5. Statistical analyses

Data were ordered in homogeneous sets, and the Student’s *t* test of independence was applied [41].

## 4. Conclusions

Monoterpenes were the most abundant components of all the oils analysed, except for the fennel and anise oils. In particular, a high presence of oxygenated monoterpenes is related to a potent phytotoxic activity. Vokou and coworkers [21] studied the allelopathic activities of 47 monoterpenoids belonging to different chemical groups, estimating their effects on seed germination and subsequent growth of *Lactuca sativa* seedlings and found that the most active compounds against both processes belonged to the groups of ketones and alcohols, followed by the group of aldehydes and phenols. Our data agree with this finding: all oils were active against germination and early radicle growth of *Lepidium sativum, Raphanus sativus* and *Lactuca sativa,* but at different levels of activity. On the other hand, it appears confirmed, by our data *in vitro*, the potent biological activities of essential oils from aromatic plants of the Mediterranean ecosystem [9].

Future experiments, involving both essential oils and each of their components, could focus on the possible effects of the length of time during which such compounds are present in soil, possible structural modifications with consequent loss or acquisition of activity, and biological action on weed seeds in field conditions. However, the specific structural factors, that operate and determine the activity of monoterpenoid and essential oils, remain still obscure.

## Figures and Tables

**Table 1:** Chemical composition of the studied essential oils.

Compound	Ki^a^	Ki^b^	Anise %^c^	Balm %	Basil %	Caraway %	Fennel %	Hyssop %	Lavender %	Marjoram %	Oregano %	Sage %	Thyme %	Vervain %	Identification ^d^
α-Thujene	930	1035	---	0.1±0.0	T	0.2 ± 0.0	T	0.4 ± 0.0	0.2 ± 0.0	0.1 ± 0.0	0.5 ± 0.0	0.4 ± 0.0	T	---	1, 2
α-Pinene	938	1032	0.3 ± 0.0	0.9 ± 0.0	0.3 ± 0.0	0.5 ± 0.2	1.8 ± 0.1	1.0 ± 0.0	---	9.0 ± 0.1	0.4 ± 0.0	4.4 ± 0.1	2.5 ± 0.1	0.2 ± 0.0	1, 2, 3
(-)-Camphene	953	1076	---	---	---	---	---	0.2 ± 0.0	0.7 ± 0.0	0.3 ± 0.0	0.2 ± 0.0	4.1 ± 0.0	1.0 ± 0.1	---	1, 2, 3
Sabinene	973	1132	T	T	0.3 ± 0.0	1.0 ± 0.1	T	1.4 ± 0.9	T	1.1 ± 0.1	T	0.4 ± 0.0	T	0.5 ± 0.0	1, 2, 3
Hepten-3-one	975		---	T	---	---	---	---	---	T	---	---	---	0.2 ± 0.1	1, 2
β-Pinene	978	1118	---	0.4 ± 0.1	0.5 ± 0.0	7.4 ± 0.4	0.5 ± 0.1	18.2 ± 0.0	---	3.8 ± 0.9	0.2 ± 0.0	2.5 ± 0.1	---	T	1, 2, 3
*cis*-Pinane	980	1073	---	---	0.1 ± 0.0	0.1 ± 0.0	---	---	0.1 ± 0.0	---	0.1 ± 0.0	---	---	---	1, 2
Verbenene	982	1131	---	T	T	T	T	0.1 ± 0.0	T	T	T	T	T	---	1, 2
Myrcene	993	1174	---	0.1 ± 0.0	0.3 ± 0.1	0.7 ± 0.1	0.2 ± 0.1	1.8 ± 0.2	0.3 ± 0.0	0.7 ± 0.3	0.5 ± 0.0	0.5 ± 0.1	0.1 ± 0.0	---	1, 2, 3
α-Phellandrene	995	1176	0.1 ± 0.0	T	T	T	0.3 ± 0.0	T	0.2 ± 0.0	0.2 ± 0.0	0.1 ± 0.0	T	T	---	1, 2, 3
Δ3-Carene	997	1153	0.1 ± 0.0	---	---	---	0.3 ± 0.1	---	0.3 ± 0.1	0.3 ± 0.0	0.2 ± 0.0	---	---	---	1, 2, 3
α-Terpinene	1012	1188	---	0.1 ± 0.1	T	T	T	0.2 ± 0.1	T	0.1 ± 0.0	0.5 ± 0.0	T	0.1 ± 0.0	T	1, 2, 3
*o*-Cymene	1020	1187	0.1 ± 0.0	2.3 ± 0.9	0.1 ± 0.0	0.2 ± 0.0	0.7 ± 0.1	0.2 ± 0.0	0.6 ± 0.1	2.6 ± 0.9	41.9 ± 0.1	2.5 ± 0.2	56.2 ± 0.2	0.1 ± 0.0	1, 2, 3
*p*-Cymene	1024	1280	---	0.6 ± 0.0	---	0.1 ± 0.1	0.3 ± 0.0	---	0.3 ± 0.0	0.4 ± 0.1	0.1 ± 0.0	1.2 ± 0.1	0.1 ± 0.0	---	1, 2, 3
β-Phellandrene	1029	1218	T	0.3 ± 0.0	0.3 ± 0.0	0.6 ± 0.2	0.4 ± 0.1	1.8 ± 0.2	0.1 ± 0.0	9.1 ± 0.5	0.1 ± 0.0	1.0 ± 0.0	0.2 ± 0.1	0.7 ± 0.2	1, 2, 3
Limonene	1030	1203	---	1.4 ± 0.3	0.4 ± 0.0	14.3 ± 0.5	1.5 ± 0.5	1.3 ± 0.7	0.3 ± 0.0	6.4 ± 0.5	0.3 ± 0.0	1.4 ± 0.0	0.6 ± 0.0	2.3 ± 0.9	1, 2, 3
1,8-Cineole	1034	1213	---	0.2 ± 0.0	0.5 ± 0.1	0.1 ± 0.0	T	0.2 ± 0.0	T	33.5 ± 0.3	0.6 ± 0.1	4.2 ± 0.3	T	0.4 ± 0.1	1, 2
(Z)-β-Ocimene	1038	1246	T	T	0.1 ± 0.0	0.1 ± 0.0	T	0.3 ± 0.0	1.7 ± 0.3	0.1 ± 0.0	T	T	T	T	1, 2, 3
(E)-β-Ocimene	1049	1280	---	T	1.2 ± 0.0	0.3 ± 0.1	T	1.0 ± 0.0	0.6 ± 0.1	0.2 ± 0.1	T	T	T	0.3 ± 0.1	1, 2, 3
γ-Terpinene	1057	1255	T	0.4 ± 0.0	T	T	0.1 ± 0.0	0.2 ± 0.0	T	0.8 ± 0.3	2.8 ± 0.2	0.1 ± 0.0	0.4 ± 0.0	0.1 ± 0.0	1, 2, 3
*cis*-Sabinene hydrate	1063	1556	---	---	---	---	---	---	0.3 ± 0.0	---	0.2 ± 0.0	0.1 ± 0.0	---	---	1, 2, 3
*cis*-Linalol oxide	1065	1450	---	---	---	---	---	---	0.4 ± 0.1	---	---	---	---	---	1, 2, 3
Fenchone	1067	1392	0.2 ± 0.0	---	0.4 ± 0.1	---	14.2 ± 0.4	---	---	---	---	---	---	---	1, 2
Terpinolene	1086	1265	T	0.1 ± 0.0	0.1 ± 0.1	T	T	0.2 ± 0.0	T	0.2 ± 0.1	0.1 ± 0.0	T	0.7 ± 0.1	T	1, 2
Linalol	1097	1553	0.4 ± 0.1	0.7 ± 0.1	0.7 ± 0.0	0.5 ± 0.1	T	1.0 ± 0.1	23.1 ± 0.2	9.8 ± 0.7	0.7 ± 0.3	1.1 ± 0.06	0.4 ± 0.1	0.1 ± 0.0	1, 2, 3
*endo*-Fenchol	1098	1120	---	---	0.2 ± 0.0	---	---	---	---	---	---	---	---	---	1, 2
*cis*-Thujone	1105	1430	---	T	---	---	---	0.1 ± 0.0	T	---	T	---	T	---	1, 2, 3
*trans*-Thujone	1115	1449	---	---	---	0.1 ± 0.0	T	---	---	T	---	37.9 ± 0.1	---	---	1, 2, 3
*trans*-Pinocarveol	1138	1654	---	---	T	T	T	0.1 ± 0.0	T	0.1 ± 0.0	T	0.2 ± 0.0	T	T	1, 2
(-)-Citronellal	1143	1491	---	39.6 ± 0.4	---	---	---	---	---	---	---	0.2 ± 0.0	0.5 ± 0.1	---	1, 2, 3
*iso*-Borneol	1144	1633	---	0.5 ± 0.0	---	---	---	---	---	0.1 ± 0.0	---	---	0.1 ± 0.0	---	1, 2, 3
Camphor	1145	1532	---	1.1 ± 0.0	0.6 ± 0.0	T	T	---	0.9 ± 0.0	0.2 ± 0.0	T	13.9 ± 0.7	T	---	1, 2, 3
Menthofuran	1150	1502	---	---	---	---	---	0.3 ± 0.0	---	---	---	---	---	---	1, 2, 3
*iso*-Pinocamphone	1153	1566	---	T	35.1 ± 0.0	T	T	29.1 ± 0.0	0.1 ± 0.0	0.2 ± 0.0	0.1 ± 0.0	0.1 ± 0.0	T	0.2 ± 0.0	1, 2
*trans*-Pinocamphone	1159	1160	---	0.4 ± 0.0	T	4.3 ± 0.9	---	11.2 ± 0.9	---	T	T	0.3 ± 0.0	T	T	1, 2
Lavandulol	1162	1674	---	---	---	---	---	4.4 ± 0.4	---	---	---	---	---	---	1, 2
*iso*-Menthone	1163	1503	---	8.8 ± 0.9	---	---	---	---	---	---	---	---	0.1 ± 0.0	---	1, 2, 3
Pinocarvone	1165	1587	---	T	0.4 ± 0.0	---	---	0.5 ± 0.0	T	T	T	T	T	T	1, 2
Borneol	1167	1719	---	0.1 ± 0.0	0.2 ± 0.0	---	---	0.1 ± 0.0	6.3 ± 0.9	2.0 ± 0.5	0.3 ± 0.0	7.6 ± 0.4	0.2 ± 0.0	0.1 ± 0.0	1, 2, 3
Terpinen-4-ol	1176	1611	---	0.1 ± 0.0	0.2 ± 0.0	T	T	0.3 ± 0.1	0.2 ± 0.0	0.4 ± 0.1	0.4 ± 0.0	0.5 ± 0.0	T	0.2 ± 0.0	1, 2, 3
dihydro-Carveol	1177	1755	---	---	---	---	0.3 ± 0.1	1.2 ± 0.1	0.4 ± 0.0	0.8 ± 0.1	---	0.2 ± 0.0	0.2 ± 0.0	---	1, 2
*p*-Cymen-8-ol	1185	1864	---	T	---	---	T	T	0.3 ± 0.0	0.1 ± 0.0	0.2 ± 0.0	0.1 ± 0.0	T	T	1, 2
α-Terpineol	1189	1706	T	0.1 ± 0.0	1.3 ± 0.3	T	---	1.2 ± 0.1	0.4 ± 0.0	0.7 ± 0.1	T	0.3 ± 0.0	0.3 ± 0.0	0.3 ± 0.1	1, 2, 3
Myrtenal	1193	1648	---	---	1.0 ± 0.0	0.1 ± 0.0	0.1 ± 0.0	1.0 ± 0.3	0.4 ± 0.1	0.7 ± 0.1	---	0.2 ± 0.0	0.3 ± 0.0	---	1, 2
Estragole	1195	1670	---	---	---	65.0 ± 0.9	0.8 ± 0.1	0.4 ± 0.0	---	0.1 ± 0.0	0.1 ± 0.0	T	---	---	1, 2, 3
Myrtenol	1196	1804	---	---	0.6 ± 0.0	---	---	1.3 ± 0.5	0.4 ± 0.0	0.2 ± 0.1	---	0.2 ± 0.0	0.3 ± 0.0	---	1, 2
Citronellol	1213	1772	---	6.2 ± 0.3	---	---	---	---	---	---	---	---	---	---	1, 2, 3
*cis*-Carveol	1226	1878	---	---	0.1 ± 0.0	---	---	---	---	---	---	---	---	---	1, 2
Isobornyl formate	1228	1596	---	---	---	---	---	---	---	---	---	---	---	45.4 ± 0.9	1, 2
Carvone	1241	1752	---	---	39.7 ± 0.9	---	---	---	---	---	---	---	---	---	1, 2, 3
Linalyl acetate	1248	1565	---	2.3 ± 0.3	0.4 ± 0.0	---	---	0.3 ± 0.0	44.4 ± 0.7	3.3 ± 0.6	0.1 ± 0.0	1.5 ± 0.2	---	---	1, 2, 3
Geraniol	1255	1857	---	5.7 ± 0.3	---	---	---	---	9.3 ± 0.3	0.6 ± 0.1	---	0.3 ± 0.0	---	---	1, 2
*cis*-Anethole	1262	1780	97.1 ± 0.4	---	---	T	76.3 ± 0.9	0.3 ± 0.0	---	---	---	---	---	0.2 ± 0.0	1, 2
(E)-Citral	1270	1727	---	---	---	---	---	---	---	---	---	---	---	44.5 ± 0.9	1, 2, 3
Isobornyl acetate	1277		---	T	T	0.1 ± 0.0	---	---	0.3 ± 0.0	0.6 ± 0.1	T	0.7 ± 0.0	T	T	1, 2
Bornyl acetate	1284	1591	---	T	T	0.1 ± 0.0	---	T	0.2 ± 0.0	1.2 ± 0.5	T	0.88 ± 0.0	T	T	1, 2
Cinnamic acid methyl ester	1289		---	---	0.1 ± 0.0	---	---	---	---	---	---	---	---	---	1, 2
Thymol	1290	2198	---	0.1 ± 0.0	---	---	---	T	---	0.7 ± 0.1	0.7 ± 0.0	T	8.7 ± 0.9	---	1, 2, 3
Carvacrol	1297	2239	---	13.3 ± 0.9	T	---	T	T	---	4.1 ± 0.9	44.0 ± 0.9	0.3 ± 0.0	24.4 ± 0.9	---	1, 2, 3
Myrtenyl acetate	1313	1698	---	---	0.5 ± 0.0	T	---	0.6 ± 0.0	---	T	T	T	---	---	1, 2
Terpinyl acetate	1333	1709	---	---	---	---	---	---	---	0.5 ± 0.0	---	---	---	---	1, 2
Eugenol	1353	2186	---	0.5 ± 0.0	---	---	---	---	---	---	---	---	---	---	1, 2, 3
Citronellyl acetate	1358	1662	---	1.6 ± 0.9	---	---	---	---	---	---	---	---	---	---	1, 2
Methyl eugenol	1369	2023	---	T	0.5 ± 0.0	0.6 ± 0.1	T	0.7 ± 0.0	T	---	T	---	---	T	1, 2
α-Copaene	1377	1497	---	T	0.1 ± 0.0	T	T	0.1 ± 0.0	T	0.1 ± 0.0	0.1 ± 0.0	T	T	0.2 ± 0.1	1, 2
Geranyl acetate	1379	1765	---	1.7 ± 0.3	---	---	---	---	---	---	---	---	---	---	1, 2
Isoledene	1382	1367	---	T	0.1 ± 0.0	T	T	0.1 ± 0.0	T	T	0.1 ± 0.0	T	T	0.1 ± 0.0	1, 2
β-Bourbonene	1385	1535	---	---	1.2 ± 0.3	---	---	1.3 ± 0.3	---	---	---	---	---	---	1, 2
β-Elemene	1387	1600	---	0.6 ± 0.0	0.1 ± 0.0	0.2 ± 0.0	T	T	---	T	T	---	T	0.2 ± 0.1	1, 2
α-Gurjunene	1408	1529	---	0.4 ± 0.0	0.4 ± 0.0	---	---	0.5 ± 0.0	---	---	---	---	---	---	1, 2
Longifolene	1411	1576	---	0.9 ± 0.1	0.5 ± 0.0	---	T	0.5 ± 0.0	T	0.1 ± 0.0	T	T	T	T	1, 2
β-Caryophyllene	1418	1612	T	0.6 ± 0.0	1.4 ± 0.5	0.1 ± 0.0	T	1.0 ± 0.5	1.0 ± 0.9	0.3 ± 0.1	0.2 ± 0.1	1.3 ± 0.0	0.1 ± 0.0	0.1 ± 0.1	1, 2
β-Cedrene	1424	1638	---	0.3 ± 0.0	0.5 ± 0.0	---	---	0.6 ± 0.0	1.3 ± 0.1	0.5 ± 0.1	0.6 ± 0.0	1.0 ± 0.0	---	0.4 ± 0.1	1, 2
Aromadendrene	1437	1628	T	T	T	0.2 ± 0.0	T	T	T	T	T	0.1 ± 0.0	T	---	1, 2
α-Humulene	1455	1689	---	0.2 ± 0.0	0.5 ± 0.0	T	T	0.6 ± 0.0	0.6 ± 0.0	0.3 ± 0.1	0.1 ± 0.0	5.9 ± 0.9	T	0.2 ± 0.0	1, 2
*allo*-Aromadendrene	1463	1661	---	T	1.2 ± 0.5	T	T	1.4 ± 0.2	0.5 ± 0.0	T	T	0.1 ± 0.0	T	0.1 ± 0.0	1, 2
Neryl isobutyrate	1468	1870	---	---	---	---	---	---	0.1 ± 0.0	---	---	---	---	---	1, 2
γ-Gurjunene	1473	1687	---	0.2 ± 0.0	0.5 ± 0.0	---	T	0.1 ± 0.0	---	0.1 ± 0.0	0.1 ± 0.0	0.1 ± 0.0	T	T	1, 2
*cis*-*β*-Guaiene	1490	1694	---	0.1 ± 0.0	---	0.4 ± 0.2	---	0.4 ± 0.0	---	---	---	---	---	---	1, 2
Bicyclogermacrene	1491	1756	---	---	1.5 ± 0.0	T	---	3.1 ± 0.5	---	0.1 ± 0.0	---	---	---	0.1 ± 0.0	1, 2
*cis*-Muurola-4(14),5-diene	1510	1675	---	2.3 ± 0.5	3.0 ± 0.9	0.1 ± 0.0	T	3.7 ± 0.9	0.3 ± 0.0	0.1 ± 0.0	T	T	T	0.2 ± 0.1	1, 2
α-7-*epi*-Selinene	1518	1740	---	0.6 ± 0.0	0.1 ± 0.0	T	T	0.1 ± 0.0	---	0.1 ± 0.0	0.1 ± 0.0	0.1 ± 0.0	T	0.2 ± 0.1	1, 2
Caryophyllene oxide	1580	2008	---	0.2 ± 0.0	---	---	---	---	0.4 ± 0.0	---	0.2 ± 0.0	0.8 ± 0.0	---	---	1, 2
α-Cadinol	1652	2255	---	0.2 ± 0.0	---	0.6 ± 0.1	---	0.3 ± 0.0	---	---	---	---	---	---	1, 2
Total compounds			**98.3**	**96.3**	**97.3**	**98**	**97.8**	**96.4**	**97.0**	**97**	**96.9**	**98.7**	**97.5**	**97.6**	
Monoterpene hydrocarbons			0.6	6.7	3.7	25.5	6.1	28.3	5.4	35.4	48	18.5	61.9	4.2	
Oxygenated Monoterpenes			0.6	83	82.4	70.9	15.4	54	87.2	59.9	47.4	70.1	35.5	91.2	
Sesquiterpene hydrocarbons			0	6.2	11.1	1	0	13.5	3.7	1.7	1.3	8.6	0.1	1.8	
Oxygenated Sesquiterpenes			0	0.4	0	0.6	0	0.3	0.4	0	0.2	0.8	0	0	
Non terpenes			97.1	0	0.2	0	76.3	0.3	0	0	0	0	0	0.4	

The analyses were carried out in triplicate; ^a^: Kovats retention index on HP-5 MS column; ^b^: Kovats retention index on HP Innowax; ^c^ --- = absent; t = trace, less than 0.05%; ^d^: Identification based on: 1 = Kovats retention index, 2 = mass spectrum, 3 = coinjection with authentic compound.

**Table 2:** Percentage composition of twelve essential oils on the basis of their chemical groups.

Plant	Alcohols	Aldehydes	Alkenes	Ketones	Phenols
**Anise**	0.4	0	0.5	0.2	97.1
**Balm**	13.9	39.6	10.2	10.3	13.4
**Basil**	3.3	1	14.6	76.2	0
**Caraway**	0.5	0.1	26.1	4.4	65
**Fennel**	0.3	0.1	5.1	14.2	77.1
**Hyssop**	9.6	1	41.6	40.9	0.7
**Lavender**	41.1	0.4	8.5	1	0
**Marjoram**	14.8	0.7	34	0.4	4.9
**Oregano**	1.8	0	7.4	0.1	44.8
**Sage**	10.6	0.4	24.2	52.2	0.3
**Thyme**	1.5	0.8	5.7	0.1	33.1
**Vervain**	0.7	44.5	5.7	0.4	0.2

**Table 3:** Effects of different doses of essential oils on germination and radicle elongation of *Lepidium sativum*. The data are expressed as mean of three replicates ± SE.

**Germination (number of seeds)**												
**Control (μg/mL)**	**Anise**	**Balm**	**Basil**	**Caraway**	**Fennel**	**Hyssop**	**Lavender**	**Marjoram**	**Oregano**	**Sage**	**Thyme**	**Vervain**
	9.3 ± 0.6	9.3 ± 0.6	9.3 ± 0.6	9.3 ± 0.6	9.3 ± 0.6	9.3 ± 0.6	9.3 ± 0.6	9.3 ± 0.6	9.3 ± 0.6	9.3 ± 0.6	9.3 ± 0.6	9.3 ± 0.6
**0.06**	10.0 ± 0.0	9.7 ± 0.6	10.0 ± 0.0	9.7 ± 0.6	9.7 ± 0.6	9.3 ± 1.2	8.7 ± 0.6	9.0 ± 1.0	9.3 ± 0.6	9.0 ± 1.0	9.7 ± 0.6	9.3 ± 0.6
**0.125**	10.0 ± 0.0	9.0 ± 0.0	9.3 ± 0.6	8.3 ± 0.6	9.7 ± 0.6	9.7 ± 0.6	9.3 ± 0.6	9.0 ± 1.0	8.3 ± 2.1	9.0 ± 1.0	9.7 ± 0.6	8.3 ± 1.2
**0.25**	8.7 ± 0.6	9.0 ± 1.7	9.7 ± 0.6	8.3 ± 1.2	9.3 ± 0.6	10.0 ± 0.0	9.3 ± 0.6	8.7 ± 1.2	6.3 ± 4.7	9.7 ± 0.6	7.7 ± 0.6*	0.0 ± 0.0***
**0.625**	9.7 ± 0.6	5.3 ± 1.2***	9.7 ± 0.6	3.7 ± 2.1*	9.3 ± 0.6*	9.3 ± 0.6	9.7 ± 0.6	10.0 ± 0.0	7.3 ± 2.1	9.3 ± 0.6	4.0 ± 1.7**	9.0 ± 1.0
**1.25**	8.0 ± 1.0	0.3 ± 0.6***	8.3 ± 1.2	0.7 ± 1.2***	9.0 ± 1.0	8.7 ± 0.6	8.3 ± 1.2	8.7 ± 0.6	0.0 ± 0.0***	8.3 ± 1.5	0.0 ± 0.0***	8.7 ± 0.6
**2.5**	0.7 ± 1.2***	0.0 ± 0.0***	3.3 ± 3.5*	0.0 ± 0.0***	4.3 ± 2.3*	0.0 ± 0.0***	1.3 ± 2.3**	6.3 ± 1.5*	0.3 ± 0.6***	1.3 ± 1.5**	0.0 ± 0.0***	0.0 ± 0.0***
**Radicle growth (length of seeds)**												
**Control** **(μg/mL)**	**Anise**	**Balm**	**Basil**	**Caraway**	**Fennel**	**Hyssop**	**Lavender**	**Marjoram**	**Oregano**	**Sage**	**Thyme**	**Vervain**
	6.1 ± 1.3	6.1 ± 1.3	6.1 ± 1.3	6.1 ± 1.3	6.1 ± 1.3	6.1 ± 1.3	6.1 ± 1.3	6.1 ± 1.3	6.1 ± 1.3	6.1 ± 1.3	6.1 ± 1.3	6.1 ± 1.3
**0.06**	7.6 ± 0.3	4.5 ± 0.4	3.7 ± 0.5*	3.4 ± 0.4*	5.7 ± 0.5	5.9 ± 1.3	4.5 ± 0.3	2.3 ± 0.5*	3.8 ± 0.9	3.9 ± 0.3*	4.3 ± 0.1	3.8 ± 0.4*
**0.125**	5.3 ± 0.1	4.2 ± 0.5	6.1 ± 0.8	2.3 ± 0.1**	6.0 ± 0.5	4.6 ± 0.4	5.1 ± 0.2	1.6 ± 0.3**	2.6 ± 0.2*	2.9 ± 0.3*	3.0 ± 0.4*	1.9 ± 0.5**
**0.25**	3.4 ± 0.6*	1.0 ± 0.7**	4.5 ± 0.9	1.9 ± 0.3**	4.9 ± 0.3	3.5 ± 0.3*	2.1 ± 0.4**	1.4 ± 0.5**	1.0 ± 0.8**	2.3 ± 0.4**	1.1 ± 0.2**	0.0 ± 0.0**
**0.625**	4.4 ± 0.9	0.4 ± 0.2**	5.8 ± 0.7	0.3 ± 0.1**	5.0 ± 0.7	3.7 ± 0.4*	3.7 ± 0.5*	1.9 ± 0.3**	0.9 ± 0.3**	0.9 ± 0.2**	0.2 ± 0.1**	2.8 ± 0.4*
**1.25**	3.1 ± 0.2*	0.0 ± 0.1**	3.4 ± 0.3*	0.1 ± 0.1**	3.4 ± 1.1	2.8 ± 0.3*	3.1 ± 0.3*	0.9 ± 0.3**	0.0 ± 0.0**	0.4 ± 0.1**	0.0 ± 0.0**	1.1 ± 0.2**

The values, followed by * (*p < 0.05; ** p < 0.01, *** p < 0.001), are statistically different according to the Student’s *t* test.

**Table 4:** Effects of different doses of essential oils on germination and radicle elongation of *Raphanus sativus*. The data are expressed as mean of three replicates ± SE.

**Germination (number of seeds)**												
**Control (μg/mL)**	**Anise**	**Balm**	**Basil**	**Caraway**	**Fennel**	**Hyssop**	**Lavender**	**Marjoram**	**Oregano**	**Sage**	**Thyme**	**Vervain**
	9.1 ± 1.1	9.1 ± 1.1	9.1 ± 1.1	9.1 ± 1.1	9.1 ± 1.1	9.1 ± 1.1	9.1 ± 1.1	9.1 ± 1.1	9.1 ± 1.1	9.1 ± 1.1	9.1 ± 1.1	9.1 ± 1.1
**0.06**	9.3 ± 0.6	7.7 ± 2.1	8.7 ± 1.5	2.7 ± 2.1***	8.7 ± 1.2	6.0 ± 0.0***	8.7 ± 1.5	8.3 ± 0.6	8.3 ± 0.6	6.0 ± 2.6**	8.0 ± 1.0	0.7 ± 1.2***
**0.125**	8.7 ± 0.6	7.7 ± 0.6	9.3 ± 1.2	0.0 ± 0.0***	8.3 ± 0.6	2.3 ± 0.6***	6.0 ± 1.7**	8.7 ± 0.6	1.0 ± 1.0***	6.7 ± 1.5**	6.7 ± 1.2**	0.0 ± 0.0***
**0.25**	9.0 ± 0.0	2.0 ± 1.0***	8.0 ± 1.0	3.3 ± 5.8**	7.3 ± 1.2*	0.0 ± 0.6***	5.0 ± 0.0***	6.3 ± 2.3**	0.0 ± 0.0***	4.7 ± 1.2***	1.3 ± 1.2***	0.0 ± 0.0***
**0.625**	8.7 ± 0.6	0.0 ± 0.0***	8.3 ± 1.2	0.0 ± 0.0***	7.0 ± 1.0**	0.7 ± 1.2***	0.0 ± 0.0***	5.7 ± 1.5***	0.0 ± 0.0***	2.0 ± 2.0***	0.0 ± 0.0***	0.0 ± 0.0***
**1.25**	9.0 ± 1.0	0.0 ± 0.0***	8.0 ± 1.7	0.0 ± 0.0***	5.7 ± 2.3**	0.0 ± 0.0***	0.0 ± 0.0***	3.3 ± 2.3***	0.0 ± 0.0***	0.7 ± 0.6***	0.0 ± 0.0***	0.0 ± 0.0***
**2.5**	8.0 ± 1.7	0.0 ± 0.0***	5.7 ± 2.5**	0.0 ± 0.0***	5.0 ± 2.0***	0.0 ± 0.0***	0.0 ± 0.0***	0.0 ± 0.0***	0.0 ± 0.0***	0.0 ± 0.0***	0.0 ± 0.0***	0.0 ± 0.0***
**Radicle growth (length of seeds)**												
**Control (μg/mL)**	**Anise**	**Balm**	**Basil**	**Caraway**	**Fennel**	**Hyssop**	**Lavender**	**Marjoram**	**Oregano**	**Sage**	**Thyme**	**Vervain**
	3.0 ± 0.9	3.0 ± 0.9	3.0 ± 0.9	3.0 ± 0.9	3.0 ± 0.9	3.0 ± 0.9	3.0 ± 0.9	3.0 ± 0.9	3.0 ± 0.9	3.0 ± 0.9	3.0 ± 0.9	3.0 ± 0.9
**0.06**	3.2 ± 0.2	2.5 ± 0.3	2.9 ± 0.1	1.5 ± 0.6*	2.7 ± 0.8	1.6 ± 0.4*	2.7 ± 0.3	2.3 ± 0.4	1.6 ± 0.3*	2.2 ± 0.4	1.7 ± 0.7*	0.2 ± 0.3***
**0.125**	3.1 ± 0.1	1.8 ± 0.4	2.8 ± 0.6	0.0 ± 0.0***	2.3 ± 0.3	1.4 ± 0.7*	2.5 ± 0.2	2.8 ± 0.3	0.5 ± 0.5***	2.1 ± 0.3	0.8 ± 0.2**	0.0 ± 0.0***
**0.25**	2.9 ± 0.2	0.3 ± 0.2***	2.6 ± 0.1	0.3 ± 0.6***	2.9 ± 1.4	0.9 ± 1.3**	2.3 ± 0.2	2.1 ± 0.6	0.0 ± 0.0***	1.6 ± 0.4*	0.7 ± 0.6***	0.0 ± 0.0***
**0.625**	2.7 ± 0.2	0.0 ± 0.0***	2.3 ± 0.4	0.0 ± 0.0***	2.3 ± 0.4	0.4 ± 0.7***	0.0 ± 0.0***	1.7 ± 0.2*	0.0 ± 0.0***	0.9 ± 0.8**	0.0 ± 0.0***	0.0 ± 0.0***
**1.25**	2.0 ± 0.1	0.0 ± 0.0***	2.7 ± 0.5	0.0 ± 0.0***	1.9 ± 0.3	0.0 ± 0.0***	0.0 ± 0.0***	1.3 ± 0.4**	0.0 ± 0.0***	0.5 ± 0.5***	0.0 ± 0.0***	0.0 ± 0.0***

The values, followed by * (*p < 0.05; ** p < 0.01, *** p < 0.001), are statistically different according to the Student’s *t* test.

**Table 5:** Effects of different doses of essential oils on germination and radicle elongation of *Lactuca sativa*. The data are expressed as mean of three replicates ± SE.

**Germination (number of seeds)**												
**Control (μg/mL)**	**Anise**	**Balm**	**Basil**	**Caraway**	**Fennel**	**Hyssop**	**Lavender**	**Marjoram**	**Oregano**	**Sage**	**Thyme**	**Vervain**
	5.6 ± 1.5	5.6 ± 1.5	5.6 ± 1.5	5.6 ± 1.5	5.6 ± 1.5	5.6 ± 1.5	5.6 ± 1.5	5.6 ± 1.5	5.6 ± 1.5	5.6 ± 1.5	5.6 ± 1.5	5.6 ± 1.5
**0.06**	6.7 ± 1.2	1.7 ± 0.6**	5.3 ± 2.5	0.0 ± 0.0***	5.3 ± 1.2	5.3 ± 1.5	4.3 ± 2.5	3.0 ± 1.7	7.7 ± 0.6	4.0 ± 2.0	0.0 ± 0.0***	0.3 ± 0.6**
**0.125**	6.3 ± 1.2	1.0 ± 1.0**	5.7 ± 1.5	2.3 ± 0.6*	7.7 ± 2.3	3.7 ± 1.2	5.0 ± 2.6	5.0 ± 1.7	3.7 ± 2.9	1.3 ± 1.5**	0.0 ± 0.0***	0.7 ± 1.2**
**0.25**	8.7 ± 1.2*	0.0 ± 0.0***	3.0 ± 1.7	0.3 ± 0.6**	6.7 ± 2.1	3.7 ± 1.5	2.7 ± 1.2*	4.7 ± 0.6	0.3 ± 0.6**	2.0 ± 1.0*	0.0 ± 0.0***	1.0 ± 1.7**
**0.625**	5.7 ± 0.6	0.0 ± 0.0***	4.0 ± 1.0	0.3 ± 0.6**	7.7 ± 1.5	3.3 ± 3.2	0.3 ± 0.6**	1.3 ± 0.6**	0.0 ± 0.0***	0.3 ± 0.6**	0.0 ± 0.0***	0.0 ± 0.0***
**1.25**	8.7 ± 0.6	0.0 ± 0.0***	4.7 ± 0.6	0.0 ± 0.0***	6.0 ± 2.6	4.7 ± 2.1	0.7 ± 0.6**	0.0 ± 0.0***	0.0 ± 0.0***	1.0 ± 1.7**	0.0 ± 0.0***	0.0 ± 0.0***
**2.5**	1.0 ± 1.0*	0.0 ± 0.0***	1.7 ± 2.1*	0.0 ± 0.0***	5.3 ± 3.1	0.0 ± 0.0***	0.7 ± 0.6**	0.0 ± 0.0***	0.0 ± 0.0***	0.3 ± 0.6**	0.0 ± 0.0***	0.0 ± 0.0***
**Radicle growth (length of seeds)**												
**Control (μg/mL)**	**Anise**	**Balm**	**Basil**	**Caraway**	**Fennel**	**Hyssop**	**Lavender**	**Marjoram**	**Oregano**	**Sage**	**Thyme**	**Vervain**
	1.2 ± 0.2	1.2 ± 0.2	1.2 ± 0.2	1.2 ± 0.2	1.2 ± 0.2	1.2 ± 0.2	1.2 ± 0.2	1.2 ± 0.2	1.2 ± 0.2	1.2 ± 0.2	1.2 ± 0.2	1.2 ± 0.2
**0.06**	2.0 ± 0.3**	0.7 ± 0.9	1.4 ± 0.3	0.0 ± 0.0***	1.6 ± 0.3	0.9 ± 0.3	0.9 ± 0.6	0.5 ± 0.1**	1.3 ± 0.4	0.9 ± 0.2	0.0 ± 0.0***	0.1 ± 0.1***
**0.125**	1.3 ± 0.1	0.2 ± 0.2***	0.7 ± 0.1**	0.3 ± 0.1***	1.4 ± 0.3	0.3 ± 0.1***	0.5 ± 0.2**	0.3 ± 0.1***	0.4 ± 0.1***	0.3 ± 0.3**	0.0 ± 0.0***	0.1 ± 0.1***
**0.25**	1.3 ± 0.4	0.0 ± 0.0***	0.4 ± 0.2**	0.1 ± 0.1***	0.7 ± 0.1**	0.4 ± 0.2**	0.2 ± 0.1***	0.4 ± 0.2**	0.2 ± 0.3***	0.6 ± 0.3*	0.0 ± 0.0***	0.1 ± 0.1***
**0.625**	1.1 ± 0.3	0.0 ± 0.0***	0.6 ± 0.2**	0.1 ± 0.1***	0.9 ± 0.1	0.4 ± 0.1***	0.1 ± 0.1***	0.4 ± 0.1***	0.0 ± 0.0***	0.1 ± 0.2***	0.0 ± 0.0***	0.0 ± 0.0***
**1.25**	1.8 ± 0.3*	0.0 ± 0.0***	0.5 ± 0.1**	0.0 ± 0.0***	0.7 ± 0.1**	0.2 ± 0.1***	0.2 ± 0.2***	0.2 ± 0.1***	0.0 ± 0.0***	0.1 ± 0.1***	0.0 ± 0.0***	0.0 ± 0.0***

The values, followed by * (*p < 0.05; ** p < 0.01, *** p < 0.001), are statistically different according to the Student’s *t* test.
